# Modulation of the immune response by helminths: a role for serotonin?

**DOI:** 10.1042/BSR20180027

**Published:** 2018-09-21

**Authors:** Susan J. Wang, Keith A. Sharkey, Derek M. McKay

**Affiliations:** 1 Gastrointestinal Research Group and Inflammation Research Network, Department of Physiology and Pharmacology, Calvin, Joan and Phoebe Snyder Institute for Chronic Diseases, Cumming School of Medicine, Calgary, Alberta, Canada; 2 Hotchkiss Brain Institute, University of Calgary, Calgary, Alberta, Canada

**Keywords:** enterochromaffin cell, helminth, intestine, serotonin

## Abstract

The mammalian gut is a remarkable organ: with a nervous system that rivals the spinal cord, it is the body’s largest repository of immune and endocrine cells and houses an immense and complex microbiota. Infection with helminth parasites elicits a conserved program of effector and regulatory immune responses to eradicate the worm, limit tissue damage, and return the gut to homeostasis. Discrete changes in the nervous system, and to a lesser extent the enteroendocrine system, occur following helminth infection but the importance of these adaptations in expelling the worm is poorly understood. Approximately 90% of the body’s serotonin (5-hydroxytryptamine (5-HT)) is made in enterochromaffin (EC) cells in the gut, indicative of the importance of this amine in intestinal function. Signaling via a plethora of receptor subtypes, substantial evidence illustrates that 5-HT affects immunity. A small number of studies document changes in 5-HT levels following infection with helminth parasites, but these have not been complemented by an understanding of the role of 5-HT in the host–parasite interaction. In reviewing this area, the gap in knowledge of how changes in the enteric serotonergic system affects the outcome of infection with intestinal helminths is apparent. We present this as a call-to-action by investigators in the field. We contend that neuronal EC cell–immune interactions in the gut are essential in maintaining homeostasis and, when perturbed, contribute to pathophysiology. The full affect of infection with helminth parasites needs to define, and then mechanistically dissect the role of the enteric nervous and enteroendocrine systems of the gut.

## Introduction

The traditional approach to understand biological systems is to reduce them to their components, classify them, and then discuss them in a siloed context. This undermines the complexity of tissues and organs where connections within intracellular signaling cascades and between cell types govern physiology and control responses to noxious stimuli, inert or infectious. For instance, despite the innumerable examples of bi-directional communication between nerves and immune cells (i.e. neuroimmunity), analyses of host responses to infection often focus on defining the immune response or altered neuronal activity. Some notable exceptions exist, such as pioneering work demonstrating nerve–mast cell interaction following infection with parasitic nematodes [1] and the recent finding that neuromedin U influences the function of innate lymphoid cells (ILCs) in the context of infection with nematodes [2,3].

Infection with a helminth parasite is a potent stimulus of host immunity that seeks to destroy/inactivate and eradicate the worm, while the parasite strives to circumvent the host’s anti-worm efforts, allowing establishment, access to nutrients, and completion of its’ life cycle. With respect to infection with helminth parasites, changes in the pattern of the enteric nervous system (ENS), neurotransmitter content and neurotransmission, and to a lesser extent enteroendocrine cells have been described [4], but these are seldom intercalated with host immunity. Thus, the primary goals of this commentary, using serotonin (5-hydroxytryptamine (5-HT)) as an example, are to: (i) draw attention to this gap in knowledge of host responses to infection with gastrointestinal (GI) helminths; (ii) demonstrate, at least in principle, how knowledge of serotonergic activity advances awareness of the host–parasite relationship; and, (iii) to speculate how such knowledge could be used to treat infection with helminth parasites.

Table 1 presents a summary of studies showing changes in the level of 5-HT in response to infection with helminth parasites, where few have defined the functional significance of these changes. We present an analysis of host serotonergic changes from the perspective of an anti-worm response, but the helminth is not passive in the host–parasite interaction and modification of the 5-HT signaling in the host could be interpreted as beneficial for the parasite: this needs to be borne in mind. For example, increased peristalsis in the infected gut could help dislodge and expel the worm conferring a benefit to the host, but it would also propel parasite eggs out of the host conferring benefits to the parasite.

**Table 1:** Summary of studies showing changes in GI serotonin following infection with a parasitic helminth

Group	Species	Host species	Findings	Reference
Cestoda	*Hymenolepis diminuta*	Rat	Higher 5-HT content in lumen and blood of infected rats fed 5-HT-supplemented diet compared with controls. Assessed using fluorescence spectroscopy	[103]
		Rat	Higher 5-HT levels in intestines of parasitized rats than in controls, assessed using fluorescence spectroscopy	[130]
		Mouse	Greater 5-HT^+^ EC cell numbers in ileum of parasitized mice compared with controls. Assessed by immunohistochemistry	[101]
	*Glanitaenia osculata*	Wels catfish (*Silurus glanis)*	Greater 5-HT^+^ EC cells in the intestine of infected catfish compared with controls. Assessed by immunohistochemistry	[111]
Nematoda	*Trichinella spiralis*	Mouse	Greater 5-HT^+^ EC cell density in duodenum and jejunum in infected mice 14 days post- infection compared with controls, while infection significantly reduced jejunal SERT expression. Assessed by immunohistochemistry	[102]
			Higher jejunal 5-HT content 14 and 28 days post-infection. Assessed by HPLC	[109]
			Reduced 5-HT brain levels in infected mice compared with controls	[141]
	*Trichuris muris*	Mouse	Higher 5-HT content and EC cell number in colon of mice 14 days post-infection compared with controls. Assessed by immunohistochemistry	[97]
			Greater 5-HT^+^ EC cell number and content in the colon 14 days post-infection compared with controls. Assessed by immunohistochemistry and ELISA	[105]
	*Trichostrongylus colubriformis*	Guinea pig	Increased serotonin in infected guinea pigs compared with controls	[106]
	*Nippostrongylus brasiliensis*	Rat	Greater mucosal mast cell number and mast cell 5-HT content, but no change in EC cell number or EC cell 5-HT in infected rats compared with controls. Assessed by cytofluorometric measurement of EC cells	[9]
			Greater levels of 5-HT in the small intestine of infected rats compared with controls. Assessed fluorimetrically	[110]
	*Microphallus papillorobustus*	*Gammarus insensibilis*	Reduced 5-HT immunoreactivity in the optic neuropils of infected animals compared with controls. Assessed by morphometric analysis of 5-HT immunoreactivity	[112]
Ancanthocephela	*Pomphorhynchus laevis*	Chub (*Squalius cephalus)*	Greater 5-HT^+^ EC cell number in intestines of infected compared with uninfected fish. Assessed by immunohistochemistry	[104]
		Chub (*Leuciscus cephalus)*	Greater 5-HT^+^ cells in tunica propria/submucosa of infected fish compared with controls; assessed by immunohistochemistry	[142]
	*Pomphorhynchus tereticollis*	*Gammarus pulex*	Greater brain 5-HT immunoreactivity compared with uninfected controls. Assessed by optical densitometric measures of immunoreactivity	[120]
	*Dentitruncus truttae*	Brown trout (*Salmo trutto)*	Greater intestinal mast cell and 5-HT^+^ endocrine cell number in infected brown trout compared with controls; assessed by immunohistochemistry	[107]

Abbreviations: EC, enterochromaffin; SERT, serotonin reuptake transporter; 5-HT^+^, serotonin immunoreactive cell.

## Host immune activity following infection with helminths

The host response to infection is co-ordinated and multicellular, where following detection of the parasite the host seeks to destroy/eradicate the parasite at the cost of minimal collateral tissue damage. However, co-evolution has not left the parasite unarmed as it seeks to establish in the host and reproduce. Excellent reviews on the host immune response following infection by helminths are available [5,6]; here we provide a broad overview of immunity to introduce putative targets for modulation by 5-HT.

Conceptually immunity is divided into innate and adaptive responses: the former are rapid and typically identify pathogens based on germ line-encoded receptors (e.g. Toll-like receptors) that detect immutable components of the pathogens. Adaptive immunity resides in T and B lymphocytes, improving in speed and specificity with repeated exposure to the same pathogen/antigen. On exposure to a novel pathogen, innate responses proceed and mold adaptive immunity. Immune responses can be considered as cell-mediated (e.g. macrophage phagocytosis or cytotoxic T cells) or humoral (e.g. antibody and complement).

Broadly, innate immunity consists of physical (e.g. mucus) or secreted (e.g. antimicrobial peptides) barriers and cellular activity by myeloid/granulocytes such as macrophages, dendritic cells (DC), basophils, mast cells, neutrophils, and eosinophils: all are important components of the host response to helminths. Goblet cell hyperplasia is characteristic of infection with intestinal helminths, as is water flux into the lumen [7]. Mastocytosis accompanies infection with helminths, often persisting beyond expulsion of the worm such as with *Nippostrongylus brasiliensis* or *Trichinella spiralis* [8,9]*.* The role of mast cells in expelling helminths is species-specific, and 5-HT release upon activation of rodent mast cells could be an important element of the host response to infection. Basophils have been proposed as a critical early source of interleukin (IL) 4 (IL-4) following infection with helminths that polarizes CD4^+^ T cells to a T helper 2 (Th2) phenotype, which become a major source of IL-4 to promote mastocytosis and goblet cell hyperplasia [10].

An eosinophil response, by virtue of IL-5 production, is characteristic of infection with virtually all worm parasites; yet, the role of eosinophils in the anti-worm response is unclear [11]. Mice lacking eosinophils are capable of mounting a primary response to several intestinal helminths [12–15], whereas upon secondary infection with *Heligmosomoides polygyrus, T. spiralis*, or *N. brasiliensis*, mice lacking eosinophils have an increased worm burden [11,15].

Macrophage and DC antigen-presenting function direct adaptive immunity that is crucial for worm expulsion (see below). Macrophages can also be important in granuloma formation around helminth eggs (e.g. *Schistosoma mansoni*), in attacking larval stages, and in the guise of an alternatively activated macrophage (AAM), they are important in tissue restitution following damage caused by worm migration though host tissues, nematode/trematode feeding, and cestode hooks [16].

The neutrophil, a rapid responder to infection and tissue damage, is often overlooked with respect to parasitic helminths. Data are emerging showing that neutrophils can be mobilized in response to helminths, particularly in response to juvenile worms and as a reaction to tissue damage [17,18]. Intriguingly, it has been suggested that neutrophils are critical for mobilization of murine AAMs following infection with *N. brasiliensis* and that apoptotic neutrophils promote the tissue restitution program in AAMs [19].

Recently, the ILC2 was identified as a critical early-stage effector following infection with intestinal helminths [20]. Lacking a T-cell receptor, ILCs have been classified as types 1 (Tbet^+^, IFNγ), 2 (RORα^+^, IL-4), and 3 (RORγt^+^, IL-17, IL-22), paralleling Th1, Th2, and Th17 cells, respectively, with the assumption that they fulfil the necessary Th-cell role until Th cells are mobilized. In response to infection with GI helminths, tuft cells release IL-25 that activates ILC2 to release IL-4, which may feed back on to the epithelium to evoke goblet cell hyperplasia and act as a catalyst for Th2 cell polarization [21] (the transporting epithelial cells may also be a source of IL-25 and other alarmins including IL-33 and thymic stromal lymphopoietin (TSLP)) [22]. Recently, two distinct subsets of tuft cells were identified based on expression of genes related to neurone development or for Th2 cytokine receptors [23]. The individual contributions of these cells to autocrine signaling during helminth infestation has not been determined.

Adaptive, or learned, immunity depends on the variation and specificity within the T- and B-cell receptors. While innate and adaptive immunity are linked, observations showing that rejection of helminth parasites from a non-permissive mammalian host is either absent or severely compromised in animals lacking T lymphocytes and/or IL-4/IL4-receptors have solidified the view that an effective anti-helminth response requires a functional adaptive immune response [6]. The effector mechanisms therein will be cytokines (e.g. IL-4, IL-9) from Th2 cells to mobilize and orchestrate effector mechanisms, such as antibody isotype switching to IgE and high-affinity IgG_1_, and the development of plasma cells from B cells [5]. The antibody arms granulocytes such as mast cells, can target the surface of the helminth and, via complement fixation, would contribute to damage of the helminths’ surface [5,6].

Like CD4^+^ T cells, that are subdivided based on function and cytokine production (i.e. Th1, Th2, Th9, Th17, T_reg_), B cells are heterogeneous and pleiotropic. The B1a cell is described as a source of natural antibody (e.g. IgM), whereas B1b cells contribute adaptive antibody to the immune response [24]. Regulatory B cells have also been defined based on a functional program (i.e. IL-10 production), with a lack of consensus on surface markers that define this phenotype [25]. As with all aspects of host–parasite interactions, species-specificity is a key feature of the role of B cells in response to helminths: rejection of *N. brasiliensis* was unaltered in BALB/cJhd^tm1^ mice that lack B lymphocytes, whereas expulsion of *H. polygyrus* was inhibited in this strain of mouse [26].

## Enteric 5-HT biosynthesis, uptake, release, and degradation

Typically considered a neurotransmitter, only 5% of the body’s 5-HT is produced in the mammalian brain: ~90% is made by enterochromaffin (EC) cells of the GI tract, with the remaining 5% derived from the ENS [27]. First identified as an enteramine, a vasoconstricting hormone [28], numerous biological activities have been assigned to 5-HT but the full spectrum of its function and nuanced activities remain to be determined [27].

**Figure 1:**
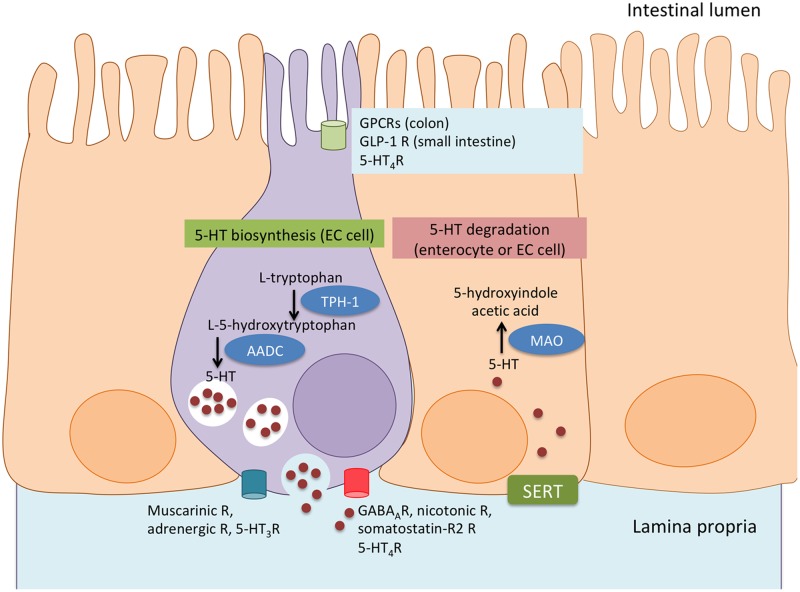
Synthesis and degradation of serotonin in EC cells Serotonin (5-HT) is synthesized by EC cells (purple) in the GI tract from l-tryptophan via the rate-limiting enzyme TPH-1. l-5-hydroxytryptophan is then converted into active 5-HT by l-aromatic acid decarboxylase (AADC) and stored in EC granules. Apically, EC cells are stimulated to secrete 5-HT by G-protein coupled receptors (GPCR) in the colon and by glucose-dependent insulinotropic peptide-1 (GLP)-1 in the small intestine, while 5-HT_4_R inhibits 5-HT release. Basolaterally, EC cells express muscarinic, adrenergic, and 5-HT_3_ receptors, activation of which leads to 5-HT release, while activation of GABA_A_, nicotinic, somatostatin-R_2_, and 5-HT_4_R inhibit 5-HT release. EC cells or enterocytes (orange) can uptake 5-HT via the serotonin reuptake transporter (SERT) and degrade 5-HT to 5-hydroxindole acetic acid via enzyme monoamine oxidase (MAO) (R, receptors).

Serotonin is produced by tryptophan hydroxylases (TPHs) that catalyze the formation of l-5-hydroxytryptophan from the essential amino acid l-tryptophan [29] (Figure 1); TPH-1 is expressed by EC cells and mast cells, macrophages, and T cells [30,31]. TPH-2 is restricted to neurones [27]. l-5-hydroxytryptophan is, in turn, transformed by l-aromatic amino acid decarboxylase into active 5-HT [29]. Recently, the gut microbiome has been shown to influence EC production of 5-HT [32]. Since intestinal helminth parasites occupy the same niche as the gut microbiota, and can affect the diversity of the microbiota [33], this raises interesting issues as to whether 5-HT synthesis is influenced by communication between helminths and microbes in the gut. This will also have implications for neuroimmune regulation of host function (discussed later).

Stored in granules and released upon stimulation by mechanical stimuli and nutrients [27,34], 5-HT plays important roles in enteric physiology. The capacity of colonic EC cells to respond to nutrients in the GI tract is attributed to their expression of G-protein coupled receptor (GPCR) sensors for microbial metabolites, such as FFAR2, OLF78, and OLF558 [34]. However, small intestinal EC cells do not possess these sensors and thus depend on paracrine interaction with glucose-dependent insulinotropic peptide-1 (GLP-1)-secreting and GPCR sensor-expressing enteroendocrine cells, through GLP-1 receptors [34]. EC cells also express adrenoreceptors, cholinergic muscarinic receptors, and 5-HT_3_ receptors, the activation of which causes 5-HT release [35]. Conversely, 5-HT release is inhibited by GABA_A_, nicotinic, somatostatin-R_2_, and 5-HT_4_ receptor activation [36]. The release of 5-HT from EC cells is mediated by Ca^2+^ to act on a variety of receptor subtypes present on cells within the GI tract [36]. Although 5-HT-containing granules are typically juxtaposed to the EC basolateral surface for release that could affect neighboring enterocytes and other cells in the mucosa, 5-HT release from the apical surface has been observed [35]. Immunocytochemistry reveals that many helminths, including those that seek to reside in the gut, such as the tapeworm *Hymenolepis diminuta*, have a serotonergic nervous system [37]. What is unclear, is whether gut parasites can co-opt 5-HT from their host (has been suggested for *H. diminuta* [38]) or if release of helminth-derived 5-HT impacts host physiology and immunity.

Due to its charged nature, 5-HT enters cells via the serotonin reuptake transporter (SERT) that is expressed on cells such as, but not limited to, neurones, EC cells, macrophages, mast cells, and DCs [39]. Platelets take up 5-HT from the gut and convey it to multiple target organs [39]. Degradation of 5-HT occurs via monoamine oxidase (MAO) to 5-hydroxyindole acetic acid [29].

## 5-HT receptor subtypes and enteric functions

Fifteen 5-HT receptors (5-HTR), classified in seven families, have been identified [40]. Of these, 5-HT_5_Rs are expressed exclusively in the central nervous system [41]. Not surprisingly, given the intestines’ capacity to make 5-HT, representatives of the other 5-HTR families occur in the gut and representatives of each family are expressed on immune cells (Table 2). The receptor subtypes most studied in the context of intestinal function are 5-HT_3_R, 5-HT_4_R, and 5-HT_7_R.

**Table 2:** Expression and function of serotonin receptor subtypes in the GI tract and immune cells

Receptor family subtype	Nature of receptor	Second messenger	Expressed on ENS	Expressed on EC	Expressed on immune cells	Common pharmacological agonists	Common pharmacological antagonists
1	G_i/o_-coupled	Reduction in cAMP levels	Yes	No	1A: monocytes/macrophages, mast cells neutrophils, B cells, T cells, 1B: DCs, eosinophils, T cells 1D: not present 1E: monocytes/macrophages, eosinophils, DCs 1F: not present	1A: 8-OH-DPAT, U-92016A F-155599 1B: L-694247, CP-94253, sumatriptan, eletriptan, 1D: PNU-109291, sumatriptan, eletriptan 1E: BRL-54443 1F: LY-344864, LY-334370, LY-573144, BRL-54443, eletriptan, sumatriptan	1A: WAY-100635, (S)-UH-301, NAD-299, NAN-190 1B: SB-236057, SB-224289, GR-55562 1D: SB-714786 1E: none 1F: none
2	G_q/11_ -coupled	Increase in IP_3_ and DAG	Yes	Yes	2A: monocytes/macrophages, DCs, eosinophils, B cells, T cells, platelets 2B: microglia, DCs, eosinophils, 2C: T cells	2A: DOI 2B: DOI, Ro-600175, BW-723C86 2C: DOI, Ro-600175, WAY-163909, locaserin	2A: Kentaserin, MDL-100907, R-96544 2B: RS-127445, EGIS-7625 2C: SB-242084, RS-102221, FR-260010
3	Ligand-gated Na^+^/K^+^ cation channel	Not applicable	Yes	Yes	Monocytes/macrophages, B cells, T cells, platelets	SR57227	Alosetron, ondansetron, granisetron
4	G_s_-coupled	Increase in cAMP levels	Yes	Yes	Monocytes/macrophages, DCs	BIMU8, ML10302, RS67506, TD-5108	Piboserod, GR-113808, SB-204070, RS-100235
5	G_i/o_-coupled	G_i/o_	No	No	5A: microglia 5B: not present	5A: none 5B: none	5A: SB-699551 5B: none
6	G_s_-coupled	G_s_	No	No	Eosinophils	WAY-181187, E-6801, EMD-386088	SB-399886, SB-271046
7	G_s_-coupled	G_s_	Yes	No	Monocytes/macrophages, microglia, DCs, B cells, T cells	E-55888, LP-44	SB-656104, SB-269970, SB-258719

Adapted from Alexander et al. (2011) [143], Nichols and Nichols (2008) [41], Beattie and Smith (2008) [144], Herr et al. (2017) [39]. Abbreviation: DAG, diacylglycerol; IP_3_, inositol triphosphate.

## 5-HT_3_R

These pentameric cation-selective ion channels are homo-oligomers consisting of 5-HT_3A_ subunits or 5-HT_3A_/5-HT_3B_ hetero-oligomers: both are expressed in the ENS [42]. The occurrence of 5-HT_3_Rs on the circular and longitudinal muscle layers of the intestinal wall and the pacemaker interstitial cells of Cajal speaks to their role in regulating motility [43]. Indeed, 5-HT_3_R activation on intrinsic afferents in the submucosal and myenteric plexuses increases motility, contributing to the peristaltic reflex in rodents [44]. 5-HT_3_Rs are also expressed on EC cells [43]. Studies with *ex vivo* preparations of mammalian small intestine reveal that activation of 5-HT_3_Rs increases 5-HT release from EC cells that is insensitive to neuronal blockade via tetrodotoxin, predicative of a positive-feedback loop in serotonergic signaling [45].

Recent studies reveal that the gut microbiota can modulate 5-HT production by EC cells [32], and that bacteria increased electrolyte secretion in mouse colon through the regulation of 5-HT_3_Rs via acetate [46]. Mice and humans infected with helminth parasites show shifts in the composition of their gut microbiota [47]: the ramifications of such for serotonergic signaling are unknown. Addressing this broad issue will shed new light on the intricacies of host–parasite interactions within the GI tract.

A third aspect of 5-HT_3_Rs function is regulation of visceral sensitivity. Luminal sensing becomes especially important when considering the presence of potentially pathogenic contents in the lumen, and the need for the body to recognize xenobiotic material and mount an appropriate defense. Interestingly, mice infected with the *N. brasiliensis*, when stressed, display reduced expression of *5-HT_3A_R* mRNA in vagal afferents [48]. Whether this translates to less pain perception or altered bowel habit that might favor establishment of the helminth is unknown.

## 5-HT_4_R

In addition to their nearly ubiquitous expression throughout the brain and contributions to cognitive processes, all six isoforms of 5-HT_4_Rs (A, B, C, D, G, I) are expressed on enteric nerves and gut smooth muscle cells where they regulate motility [49]. 5-HT_4_Rs have been identified at the luminal surface of the epithelium along the GI tract and on EC cells and goblet cells [50]. In contrast to 5-HT_3_Rs, activation of 5-HT_4_Rs on EC cells can inhibit or promote 5-HT release [50,51].

A ‘washer-sweeper’ effect often occurs in response to infection with helminth parasites [52], and 5-HT signaling would contribute to the increase in peristalsis needed for the sweeper aspect of this response. Activation of 5-HT_4_R in the gut can enhance peristalsis, and its actions are inhibitory or excitatory depending on receptor location [53]. Activation of 5-HT_4_R can result in the stimulation of excitatory cholinergic myenteric neurones leading to the release of acetylcholine [54] and substance P [55]. Enteric infection with helminths alters intestinal motility, as exemplified by increased transit in *T. spiralis*-infected rats but the degree to which serotonergic signaling, via 5-HT_3_Rs or 5-HT_4_Rs, contributes to this is unclear [56]. So what of the washer component? Water movement into the gut lumen follows vectorial ion transport (e.g. Cl^−^ secretion) [57,58]. Pharmacological studies reveal that 5-HT_4_R activation results in epithelial Cl^−^ secretion that could contribute to an inhospitable environment for helminths [59]. Another component of the washer event is mucus release: treatment with 5-HT increases goblet cells and exocytosis of mucin [60].

Adding to the list of 5-HTs biological functions, neuronal but not mucosal 5-HT can promote the development of dopaminergic myenteric enteric neurones [61], possibly via 5-HT_4_R signaling [62]. Activation of these receptors with the pharmacological agonist, tegaserod protected neurones by reducing apoptosis and promoting proliferation [62]. In addition, De Vadder et al. (2018) [63] show that the colonic microbiota can contribute to enteric neuronal maturation via the 5-HT_4_R.

## 5-HT_7_R

Three subtypes of 5-HT_7_R (A, B, and C) occur in the gut. In the guinea pig, 5-HT_7_Rs are expressed predominantly in myenteric neurones, with lesser expression in submucosal neurones and circular smooth muscle cells, and have been implicated in the regulation of circular muscle accommodation during peristalsis [64]. Emerging evidence points to 5-HT_7_R as an important regulator of inflammation in the GI tract [65,66]. For example, 5-HT_7_R is expressed on enteric neurones and on CD11c^+^ myeloid cells in the mouse colon, and is significantly increased in dextran sodium-sulphate (DSS)-induced colitis: inhibition of these receptors improved colitis [66].

## Serotonin and immunity

The GI tract is a major source of 5-HT and it has been suggested that once released into the circulation, 5-HT can be taken up by platelets for delivery to tissue remote from the gut [67]. While likely contributing a minimal amount of 5-HT to the peripheral pool, mast cells, monocytes/macrophages, and T cells can express TPH-1 [31]; these immune cells and others (e.g. DCs, platelets) also express SERT and MAO to facilitate 5-HT uptake and degradation [33,68]. The expression of nearly every subtype of 5-HTR has been observed on immune cells (Table 2).

Serotonin is a chemoattractant for human mast cells, eosinophils, and alveolar macrophages via 5-HT_1A_R, 5-HT_2A_R, and 5-HT_2C_R, respectively [69]. Serotonin influences immune cell cytokine production in a cell- and receptor-specific manner; indeed, this specificity may explain contrasting study results. As examples: (i) 5-HT_1A_R activation on murine peritoneal macrophages enhances IL-1 and IL-6 output (and phagocytosis) [70]; and, (ii) activation of 5-HT_2A_R can suppress tumor necrosis factor-α (TNF-α)-mediated inflammation [71], while NK cell synthesis of IFN-γ is increased when their 5-HT_1A_Rs are activated [72]. With human monocytes/macrophages expressing multiple 5-HTRs [68], the potential for 5-HT to modulate many or all facets of macrophage function is huge, including polarization toward the anti-inflammatory/pro-resolution M2 phenotype [73,74].

DC expression of 5-HTRs varies with their stage of maturation [75], where 5-HT_1_Rs and 5-HT_2_Rs mediate chemotaxis [76]. IL-1β and IL-8 are increased following activation of DC 5-HT_4_R and 5-HT_7_R, meanwhile release of IL-12 and TNF-α is reduced [75]; IL-6 production from DCs is up-regulated following activation of 5-HT_3_R, 5-HT_4_R, and 5-HT_7_R [75]. DCs direct differentiation of naïve T cells, and 5-HT has been implicated in this process [77], where, for example activation of 5-HT _2B_R on CD1a^+^ human monocyte-derived DCs promoted CD4^+^ T cells differentiation into Th1 and Th17 cells [78].

Serotonin receptors may have important roles in eosinophil chemotaxis [79], as in the case of 5-HT_2_Rs which have been implicated in eosinophil recruitment to the airways contributing to inflammation [80].

Linking to adaptive immunity, *para*-chlorophenylalanine (PCPA), a TPH-inhibitor, suppressed macrophage-mediated activation of T cells [81,82], and 5-HT_7_R has been shown to play a role in DC-mediated activation of the adaptive immunity [66,83]. T cells express 5-HTRs [39] (Table 2) and serotonergic signaling has been implicated in the differentiation, proliferation, and activation of T lymphocytes [84]. For example, 5-HT_2A_R and 5-HT_1C_R can mediate human T-cell proliferation stimulated by mercuric chloride [85], while 5-HT_7_Rs contribute to naive T-cell activation [86]. Activation of T cells can increase 5-HT_1B_R expression leading to Th-cell proliferation, and 5-HT_2A_R resulting in T cell differentiation and function [87,88]. B lymphocytes express SERT, and their proliferation/activation is influenced by 5-HT [68,89].

## 5-HT in enteric inflammation

The gut houses ~75% of total immune cells found in the body. Immune cells and EC cells have a close relationship, each capable of modulating the others’ function [68]: a relationship that is likely perturbed in disease. For instance, EC cells from patients with Crohn’s disease exhibit elevated 5-HT production [90], while patients with ulcerative colitis have reduced SERT expression [91]. Use of selective serotonin reuptake inhibitors has been associated with the development of microscopic colitis [92]. Mice engineered to lack components of the serotonergic system have revealed 5-HTs participation in gut disease: TPH1^−/−^ mice exhibited reduced severity of chemical-induced colitis and an accompanying reduction in IL-1β, IL-6, and TNF-α [93]. Similarly, SERT^−/−^ mice exhibited more severe colitis compared with wild-type mice [94]. In addition, Margolis et al. [95] showed that reduction in EC-derived 5-HT by the TPH inhibitors LP-920540 and LX1032 reduced the severity of trinitrobenzene sulphonic acid (TNBS)-induced colitis. Also, reduction in 5-HT production with the selective TPH-1 inhibitor, telotristat etiprate, was protective in experimental colitis [96]. This worsening of gut inflammation could, in theory, be driven by a positive-feedback loop, since both Th1 and Th2 cytokines can elicit the production and release of 5-HT from EC cells [97].

Interestingly, while EC-derived 5-HT appears to have pro-inflammatory effects, neuronal 5-HT might be anti-inflammatory in the context of experimental colitis [98]. Mice lacking TPH-2 (neuronally restricted expression) exhibit increased severity of disease accompanied by elevated TNF-α, IL-1β, and IL-6 levels during experimental colitis compared with wild-type mice. Thus, neurone-derived 5-HT may protect the ENS from inflammation [62,99], leading to increased motility which may contribute to improved disease outcomes. In addition, 5-HT_4_Rs may exert an anti-inflammatory effect in the bowel by preserving the integrity of the epithelial layer, enhancing proliferation and wound healing, and increasing the enterocytes resistance to oxidative stress [100].

## Serotonin and the modulation of anti-helminth immunity

**Figure 2:**
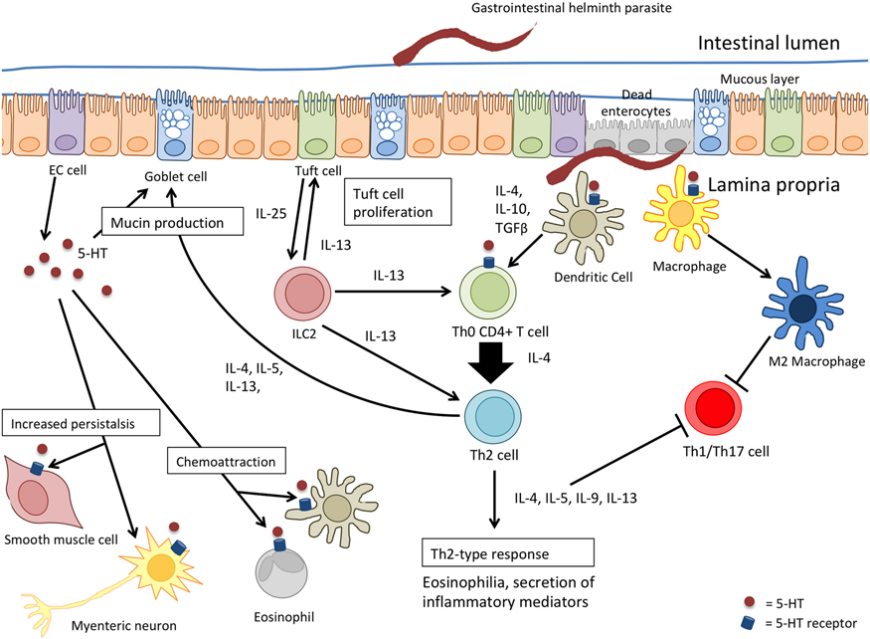
The potential for serotonin to influence the immune response against infection with helminth parasites The Th2 response following infection with helminths begins when the parasite is recognized by the epithelium (enterocyte, EC cell, or tuft cell) and/or antigen-presenting cells such as the DC or macrophages. Tuft cells release IL-25 and activate innate lymphoid type 2 cells (ILC2s). ILC2s, along with activated DCs, promote the differentiation and proliferation of naïve CD4^+^ T cells into Th2 effector cells by the release of IL-13, and then IL-4, IL-10, and TGFβ, respectively. Th2 cells subsequently secrete Th2 cytokines (IL-4, IL-5, IL-9, and IL-13) leading to responses directed at worm expulsion, such as goblet cell hyperplasia. Serotonin released by cells contributes to the ‘washer-sweeper’ response via goblet cell hyperplasia and mucin exocytosis, and increased GI peristalsis through its actions on enteric neurones and smooth muscle. The expression of serotonin receptors on the many components of the mucosal immune system suggest that 5-HT likely plays, at least, a modulatory role in the hosts’ anti-worm response.

While, changes in 5-HT levels or activity have not been a major component of investigation in host-helminth analyses, the data available show that infection with GI helminths can lead to increased enteric 5-HT content (Table 1) [9,97,101–112]. The distribution of 5-HTR on many types of immune cells that are mobilized following infection with helminth parasites affords serotonin ample opportunity to influence this host–parasite interaction (Figure 2).

The ‘washer-sweeper’ phenomenon that often accompanies infection with helminth parasites is intuitively accepted as an important anti-worm effector mechanism [56], and so the mucus and water secretagogue effect of 5-HT would be predicted to contribute to the expulsion of intestinal parasites. When reserpine was used to deplete 5-HT, this suppressed the expulsion of the nematode, *Trichostrongylus colubriformis*, from guinea pigs [113]. Contrarily, Parmentier et al. [114] found methysergide (5-HT_2B,2C_R antagonist, 5-HT_1C_R partial agonist) and ketanserin (5HT_1_R, 5HT_2_R antagonist) inhibited *T. spiralis*-induced mastocytosis and goblet cell hyperplasia in the gut but did not influence the course of parasite expulsion. Similarly, infection of rats with *N. brasiliensis* lead to a two-fold increase in enteric 5-HT, and treatment with PCPA did not alter the kinetics of worm expulsion [110]. Blocking 5-HT synthesis with telotristat etiprate, enhanced expulsion of the nematode *Trichuris muris* from mice [96]. These studies illustrate two noteworthy points. One, that specificity is the basis of host–parasite interactions and so it is expected that the contribution of 5-HT signaling to the host response to infection will be parasite- and host-specific. Second, given the number of 5-HTR subtypes, their diversity of action, cellular distribution, and density of expression, the application of 5-HT or inhibitors of its synthesis or degradation will reveal the net effect of 5-HT in the system where, conceivably, receptor subtypes with opposing bioactivities could be activated.

The hyperplasia of EC cells and mastocytosis following infection with *T. spiralis* is significantly reduced in mice lacking T cells [102], and, likewise, severe combined immunodeficient (SCID) mice that lack T and B cells infected with *T. muris* have reduced mucosal 5-HT compared with wild-type mice [105]. Following this, it was found that BALB/c mice that mobilize a robust Th2 response upon infection with *T. muris* have significantly more 5-HT and EC cell numbers compared with infected AKR mice that mobilize Th1 responses and fail to reject the worm [97]. Serotonergic responses similar to those in infected BALB/c and AKR mice were observed in STAT4^−/−^ (i.e. heightened Th2 immunity) and STAT6^−/−^ (reduced Th2 signaling) mice, respectively [97]. Collectively these studies suggest a relationship between serotonin and adaptive immunity (most prominently Th2 signaling) following infection with parasitic helminths.

The Th2 cytokine, IL-13, up-regulates expression of the 5-HT_2A_R and this was presented as part of the mechanism underlying the increase in jejunal muscle contractility in mice infected with either of the nematodes, *H. polygyrus* or *N. brasiliensis* [115]. Serotonin-induced muscle contraction *ex vivo* was significantly greater in jejunal muscle strips from *N. brasiliensis*-infected rats compared with controls [116]. This same enhanced responsiveness in jejunal muscle strips from rats infected with *T. spiralis* did not occur in athymic (T-cell deficient) rats: the 5-HT hyper-responsiveness was recapitulated in infected athymic rats reconstituted with T cells [117].

Electrophysiological analysis of the jejunum of *T. spiralis*-infected mice, where 5-HT levels were increased, revealed neuronal hyposensitivity and hypersensitivity to mechano-stimulation during acute infection and in the post-infection period (i.e. 28 days post-infection) that were mediated in part by 5-HT_3_Rs [109]. Application of worm antigen to jejunum from *T. spiralis*-infected rats mounted in Ussing chambers resulted in the release of 5-HT that evoked an active Cl^−^ secretory response via direct action on the epithelium and via nerves [7]. In contrast, others suggest that jejunum from *T. spiralis*-infected rats is hyporesponsive to 5-HT in terms of active ion transport measured in Ussing chambers [118]; this could be a consequence of receptor desensitization due to ongoing 5-HT production and release following infection. Jejunal short-circuit current (i.e. active vectorial ion transport) evoked by 5-HT is reduced in tissues from mice infected with *H. polygyrus*, an event that was dependent on IL-4 signaling, although exactly how the Th2 system mediated this change in epithelial responsiveness to 5-HT was not defined [119]. Regardless, these studies demonstrate how the adaptive immune response to infection with helminth parasites affect serotonergic regulation of intestinal muscle and epithelial function, highlighting 5-HT as a mediator bridging the innate and adaptive immune responses to helminth parasites.

## Serotonin, behavior, and immunity

While focussing on immunity, it would be remiss not to mention how helminth-evoked changes affect host behavior. This is a fascinating area and examples abound of helminth manipulation of host activity [120]. It is also clear that 5-HT has a role to play in this helminth–host interaction and a few examples of this are provided. For instance, acantocephalans (thorny-headed worms) affected behavior of their arthropod host that was associated with increased or decreased 5-HT-immunoreactivity in the hosts’ brain [121,122]. Indeed, neuro-inflammation has been postulated as an important consequence downstream of serotonergic dysfunction in infected gammarids [123]. Reduced 5-HT in the brain of killifish infected with the trematode *Euhaplorchis californiensis* was associated with abnormal stress responses [124]. Behavioral changes in *Taenia crassiceps*-infected mice correlated with a 30% reduction in 5-HT in the hippocampus [125], and, intriguingly, altered behavior in the sticklebacks infected with the tapeworm *Schistocephalus solidus* was linked to modifications in the serotonergic axis and activation of immunity [126]. The concept of bi-directional communication between the brain and the gut is established [127]; however, there is a dearth of data on how infection with helminth parasites affects this axis, the role for 5-HT therein, and the impact on the host–parasite relationship.

While beyond the scope of this review it is worth under-scoring that serotonin is but one of many host-derived factors mobilized following infection that will affect immune responsiveness and behavior, such as cortisol [128]. Reciprocally, and in a concept termed as transregulation, host-derived factors capable of affecting host immunity (e.g. dehydroepiandrosterone (DHEA), testosterone) can directly affect helminth growth and fecundity [128].

## Luminal serotonin and helminth biology

We draw attention to the possibility that there could be a direct impact on the worm: a relevant issue given data suggesting that EC cells can release 5-HT into the gut lumen [35]. Ribeiro and Webb [38] demonstrated that *H. diminuta* express a 5-HT transporter and a 5-HTR, and administration of 5-HT to infected rats resulted in increased *H. diminuta* 5-HT content [103]. Whether this is a general phenomenon relating to tapeworms or platyhelminths (the trematode, *Schistosoma mansoni*, has also been suggested to take up host-derived 5-HT [129]) is unknown, as is the fate of, and biological activity of any absorbed 5-HT. Anteriad migration of *H. diminuta* in rats has been correlated with host-feeding, which also resulted in increased luminal and worm 5-HT content, leading to the intriguing speculation that host-derived 5-HT may serve as a cue to direct the behavior (e.g. niche localization) of helminths [130].

## Helminths, bacteria, and neuroimmunity

**Figure 3:**
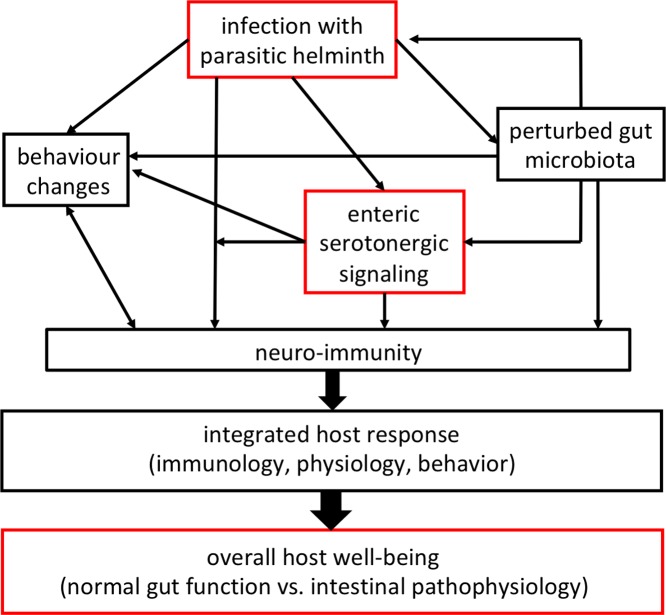
Simplified schema showing how infection with a parasitic helminth can affect serotonergic signaling to influence overall health (red boxes), and how this can be modified or mediated by the gut microbiota and integrated into a common theme of neuro-immunity

The resident bacteria of the mammalian colon can produce or consume neurotransmitters including serotonin [131], and, as noted, infection with helminths affects the composition of this microbial community [33,47]. Further, the anti-inflammatory benefit of infection with helminth parasites in mouse model systems, may be mediated, at least in part, by gut bacteria [132,133]. Reciprocally, it has been posited that helminth-evoked disturbance of the gut microbiota could affect cognition in children [134]. A recent study described abundant EC cells in Rhesus macaques with idiopathic colitis who displayed microbial dysbiosis [135]. Furthermore, bacteria and viruses can cause the release of 5-HT from EC cells that affects maturation of the ENS [63] and enteric glia cells [136]. Thus, a scenario is emerging where transkingdom interaction between helminths and bacteria influences serotonin (a representative neuroendocrine factor) biology, which in turn affects host neuroimmunity as a determinant of overall host well-being (e.g. physiology, immunology, behavior), and potentially feeds back to the helminth (Figure 3).

## Directions

The impact of 5-HT on the ENS, immune system, and enteric stromal cells is profound, complicated, and nuanced. While a few analyses have intriguingly, but not surprisingly, demonstrated increases in 5-HT in the gut following infection with helminth parasites, understanding how this biogenic amine affects the host–parasite relationship is rudimentary. However, the advent of mice engineered to lack the enzymes to synthesize 5-HT or 5-HTR subtypes (that can be knocked out in a cell-lineage specific manner), coupled to validated receptor agonists and antagonists as medications or pharmacological research reagents (Table 2) provide the opportunity for discovery and translational research. How important is 5-HT to helminth-evoked TH2 immunity? Will extrinsic pharmacological manipulation of serotonergic signaling affect helminth infectivity, fecundity, and expulsion? What, if any, is the relevance of enteric 5-HT signaling in helminth-modulation of concomitant disease? Can peripherally or luminally restricted 5-HTR agonists/antagonists be developed as new anti-helmintic strategies? The 5-HT_1c_R agonist *p*-amino-phenethyl-m-trifluoromethylphenyl piperazine (PAPP) blocked migration of the L3 stage of the nematode *Haemonchus contortus in vitro* and acted as an anti-helmintic in *H. contortus*-infected gerbils [137]. Whether the PAPP targetted the worm *in vivo* or modified host immunity was not determined, but this proof-of-concept finding suggests that targetting the host serotonergic system with peripherally restricted drugs is worth consideration.

Addressing these and a myriad of other issues, with parallel investigations of the ENS, will significantly advance awareness of neuroendocrine–immune interaction in the gut and how the integrated activity of these systems following infection determines the health status of the host.

## Conclusion

Infection with parasitic worms is a major human health concern [138], a significant impact on livestock productivity, and a common problem in companion animals. Resistance to anti-helmintics is rampant and widespread. Analyses of rodent-helminth model systems have delineated immune reactions to infection, and while assessment of the ENS in these models has lagged behind that of immune cells, there are data demonstrating perturbations in the ENS and the enteroendocrine system following infection with parasitic helminths [139]. The issue before us is the integration of these traditionally disparate systems into a holistic view of the host–parasite relationship: a co-ordinated multicellular response accomplishes the tasks of recognizing the presence of the worm and orchestrating the anti-worm response. Serotonin, in a host–parasite specific manner, is a component of the response to infection, but we lack an understanding of how 5-HT influences the immune response following infection (and *vice versa*), and if pharmacological manipulation of the serotonergic system can be exploited as an anti-helminth strategy. The advent of selective 5-HTR agonists and antagonists affords the opportunity to define precisely how this biogenic amine (as an example of a neuroendocrine factor) modulates the host–parasite interaction, as a forerunner to the intriguing possibility that peripherally restricted serotonergics could be a novel approach to the omnipresent problem of infection with helminth parasites. Finally, 5-HT expressing EC cells are only one population of enteroendocrine cells and many others have been shown to have important roles in the co-ordinated response to helminths, e.g. cholecystokinin-containing I cells [140]. Future studies aimed at fully understanding the role of the enteroendocrine system in the co-ordinated response to helminth infection would be of immense value in determining the pathophysiology of host–parasite interactions.

## Abbreviations

AAM: alternatively activated macrophage
DC: dendritic cell
EC: enterochromaffin
ENS: enteric nervous system
GI: gastrointestinal
GLP-1: glucose-dependent insulinotropic peptide-1
GPCR: G-protein coupled receptor
IFN: interferon
IL: interleukin
ILC: innate lymphoid cell
MAO: monoamine oxidase
PAPP: *p*-amino-phenethyl-m-trifluoromethylphenyl piperazine
PCPA: *para*-chlorophenylalanine
SERT: serotonin reuptake transporter
STAT: signal transdurcer and activator of transcription
Th2: T helper 2
TNF-α: tumor necrosis factor-α
TPH: tryptophan hydroxylase
T_reg_: regulatory T cell
5-HT: 5-hydroxytryptamine
5-HTR: 5-HT receptor
